# Exploring the molecular basis of *Panax notoginseng* mediated inhibition of cervical cancer through machine learning, transcriptomics analysis, network pharmacology, molecular docking, and in-silico simulation approaches

**DOI:** 10.1093/bioadv/vbag164

**Published:** 2026-07-10

**Authors:** Shah Kamal, Chen Qin, Yanjuan Wang, Ruilin He, Mohammad Amjad Kamal, Wenji Li

**Affiliations:** School of Traditional Chinese Medicine, Faculty of Medicine, Yangzhou University, Yangzhou, Jiangsu 225009, PR China; Key Laboratory of the Jiangsu Higher Education Institutions for Integrated Traditional Chinese and Western Medicine, Senile Diseases Control (Yangzhou University), Yangzhou, Jiangsu 225009, PR China; Sino-Malaysia Molecular Oncology and Traditional Chinese Medicine Delivery Joint Research Centre, Medical College, Yangzhou University, Yangzhou, Jiangsu 225009, PR China; School of Traditional Chinese Medicine, Faculty of Medicine, Yangzhou University, Yangzhou, Jiangsu 225009, PR China; Key Laboratory of the Jiangsu Higher Education Institutions for Integrated Traditional Chinese and Western Medicine, Senile Diseases Control (Yangzhou University), Yangzhou, Jiangsu 225009, PR China; Sino-Malaysia Molecular Oncology and Traditional Chinese Medicine Delivery Joint Research Centre, Medical College, Yangzhou University, Yangzhou, Jiangsu 225009, PR China; School of Traditional Chinese Medicine, Faculty of Medicine, Yangzhou University, Yangzhou, Jiangsu 225009, PR China; Key Laboratory of the Jiangsu Higher Education Institutions for Integrated Traditional Chinese and Western Medicine, Senile Diseases Control (Yangzhou University), Yangzhou, Jiangsu 225009, PR China; Sino-Malaysia Molecular Oncology and Traditional Chinese Medicine Delivery Joint Research Centre, Medical College, Yangzhou University, Yangzhou, Jiangsu 225009, PR China; School of Traditional Chinese Medicine, Faculty of Medicine, Yangzhou University, Yangzhou, Jiangsu 225009, PR China; Key Laboratory of the Jiangsu Higher Education Institutions for Integrated Traditional Chinese and Western Medicine, Senile Diseases Control (Yangzhou University), Yangzhou, Jiangsu 225009, PR China; Sino-Malaysia Molecular Oncology and Traditional Chinese Medicine Delivery Joint Research Centre, Medical College, Yangzhou University, Yangzhou, Jiangsu 225009, PR China; Future Technologies Research Centre, King Faisal University, PO Box 400, Al Ahsa 31982, Saudi Arabia; Department of Health Sciences, Novel Global Community Educational Foundation, Hebersham, NSW 2770, Australia; School of Traditional Chinese Medicine, Faculty of Medicine, Yangzhou University, Yangzhou, Jiangsu 225009, PR China; Key Laboratory of the Jiangsu Higher Education Institutions for Integrated Traditional Chinese and Western Medicine, Senile Diseases Control (Yangzhou University), Yangzhou, Jiangsu 225009, PR China; Sino-Malaysia Molecular Oncology and Traditional Chinese Medicine Delivery Joint Research Centre, Medical College, Yangzhou University, Yangzhou, Jiangsu 225009, PR China

## Abstract

**Motivation:**

Cervical cancer remains a major global health challenge, particularly in low-resource settings where treatment efficacy is limited by drug resistance and toxicity. Although *Panax notoginseng* (Sanqi) has demonstrated anticancer activity, its molecular mechanisms against cervical cancer remain insufficiently understood.

**Results:**

An integrated transcriptomic and systems pharmacology approach was employed to investigate the therapeutic mechanisms of *Panax notoginseng* in cervical cancer. Transcriptomic analysis identified 533 overlapping differentially expressed genes associated with cell cycle regulation, DNA replication, cellular senescence, and p53 signaling. Network pharmacology revealed 291 overlapping targets between *P. notoginseng* compounds and cervical cancer-related genes. Protein–protein interaction analysis identified tumor necrosis factor, interleukin-6, proto-oncogene, non-receptor tyrosine kinase (SRC), TOP2A, and CDC45 as key hub genes involved in inflammation, apoptosis, and tumor progression. Molecular docking demonstrated strong binding affinities of ginsenoside Re, Panaxadiol, Daucosterol, and Stigmasterol toward core targets, while molecular dynamics simulations confirmed stable protein–ligand interactions. Absorption, Distribution, Metabolism, Excretion, and Toxicity (ADMET) analysis suggested comparable pharmacokinetic properties and low predicted toxicity.

**Availability and implementation:**

The datasets analyzed in this study are publicly available through the Gene Expression Omnibus database under accession numbers GSE63514 and GSE9750. Additional data supporting the findings of this study are available within the article and its Supplementary Materials.

## 1 Introduction

Cervical cancer remains one of the most prevalent gynecological malignancies worldwide, particularly in low- and middle-income countries, where it significantly contributes to cancer-related mortality among women (Reza *et al.* 2024). Despite the availability of preventive vaccines and improved screening, advanced-stage cervical cancer often presents limited therapeutic options and a poor prognosis (Boitano *et al.* 2023). The pathogenesis of cervical cancer involves not only genetic and viral factors of persistent high-risk of Human papillomavirus (HPV) infection, but also chronic inflammation and aberrant activation of oncogenic signaling pathways such as IL6, tumor necrosis factor-alpha (TNF-α), and SRC kinase cascades (Pavelescu *et al.* 2025). These pathways contribute to tumor proliferation, immune evasion, angiogenesis, and therapeutic resistance, underscoring the need for more effective and multi-targeted therapeutic strategies for cervical cancer (Obeagu 2025). While conventional treatments, including chemotherapy, radiotherapy, and targeted therapies, exist, their efficacy is often compromised by resistance mechanisms, adverse side effects, and tumor heterogeneity (Liu *et al.* 2021b). In this context, Traditional Chinese Medicine (TCM) has brought in increasing attention due to its multi-target, low toxicity profiles and historical efficacy in various cancer types, including gynecological malignancies (Xi *et al.* 2025).

Among TCM herbs, *P. notoginseng* (Burk.), commonly known as (Sanqi, 三七), is a traditional Chinese medicinal herb valued for its hemostatic, anti-inflammatory, and circulatory-enhancing properties (Li *et al.* 2025a). It contains a rich repertoire of bioactive compounds, notably ginsenosides (Rg1, Rg3, Re, Rh1, Rh2), noto-ginsenosides (R1), and other phytoconstituents such as β-sitosterol, stigmasterol, daucosterol, and panaxadiol (Wei *et al.* 2024). These molecules have demonstrated modulation of apoptosis, angiogenesis, cell cycle progression, and immune response in various cancers (Mohanan *et al.* 2018). It is primarily cultivated in the Yunnan and Guangxi provinces of China (Xie and Wang 2023), and contribute to its diverse pharmacological effects. These include antioxidant, cardio-protective, neuro-protective, and anti-cancer activities (Liu *et al.* 2021b). Due to its unique phytochemical profile, it represents a promising source for novel therapeutic agents. As a major component of the *P. notoginseng* has shown growing promise in the treatment of cervical cancer, a prevalent gynecological malignancy associated with persistent HPV infection and chronic inflammation (Thorpe *et al.* 2023).

Recent studies have shown that the bioactive constituents of *P. notoginseng*, particularly ginsenosides Rg3, Rg1, and notoginsenoside R1, exert significant anticancer effects by targeting key molecular pathways involving TNF-α, SRC kinase, and interleukin-6 (IL-6). TNF-α, a pro-inflammatory cytokine elevated in cervical tumors, promotes angiogenesis, immune escape, and tumor proliferation (Wu *et al.* 2024). Ginsenoside Rg3 has been shown to suppress TNF-α expression and inhibit NF-κB signaling, thereby reducing inflammatory cytokine production and tumor growth in cervical cancer models (Yao and Zhu 2025). Similarly, SRC, a non-receptor tyrosine kinase overactivated in cervical cancer, contributes to epithelial–mesenchymal transition, metastasis, and poor prognosis (Martellucci *et al.* 2020). *Panax notoginseng* compounds have demonstrated inhibitory effects on SRC phosphorylation, thereby reducing the migratory and invasive potential of cervical cancer cells by modulating the SRC/Focal adhesion kinase (FAK)/Matrix metalloproteinase (MMP) signaling axis (Wei *et al.* 2024). Moreover, IL6, a pleiotropic cytokine linked to chronic inflammation and therapy resistance, is also downregulated by *P. notoginseng* components such as Rg1 and R1 (Mancuso 2024). This leads to reduced activation of the IL6 pathway, downregulation of anti-apoptotic genes (Bcl-2, survivin), and reprogramming of tumor-associated macrophages toward an M1 phenotype (Xu *et al.* 2022). However, the specific molecular basis and therapeutic relevance of these compounds against cervical cancer remain inadequately characterized.

**Figure 1:**
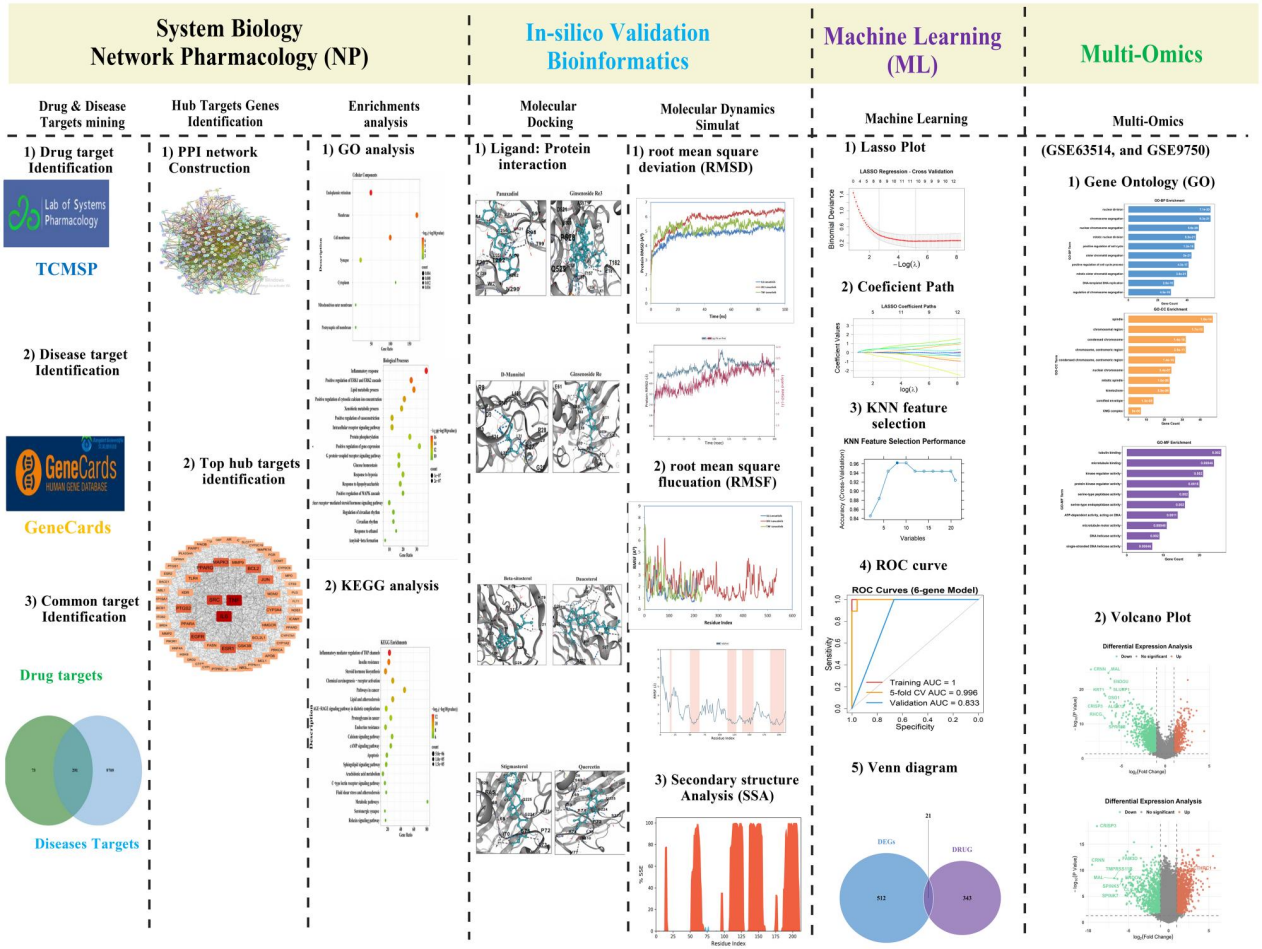
Schematic overview of the integrative computational workflow for investigating the anticancer mechanisms of *P. notoginseng* in cervical cancer. The pipeline integrates network pharmacology, bioinformatics analyses (GO/KEGG enrichment, molecular docking, and molecular dynamics), machine learning-based feature selection, and transcriptomic profiling to identify key molecular targets and pathways. (https://BioGDP.com) Online tool.

To address these challenges, we employed an integrative computational strategy combining network pharmacology, transcriptomic analysis, machine learning (ML), and molecular dynamics (MDs) simulations to systematically investigate the anticancer potential of *P. notoginseng* in cervical cancer (Fig. 1). This multi-layered framework enables the identification of bioactive compounds, prediction of molecular targets, and exploration of their functional roles within disease-associated biological networks, providing a comprehensive basis for mechanistic insight. Recent advances in multi-omics technologies, together with ML approaches, have significantly improved our understanding of cervical cancer by enabling the identification of key regulatory pathways, gene signatures, and therapeutic targets associated with tumor progression, genomic instability, and immune dysregulation.

In summary, the present study aims to characterize the key bioactive constituents of *P. notoginseng*, elucidate their molecular targets, and investigate their potential roles in modulating critical signaling pathways involved in cervical cancer progression. It provides a systematic framework for understanding the multi-target therapeutic potential of *P. notoginseng*, but further experimental validation is required to confirm these findings and establish their clinical relevance.

## 2 Methods

### 2.1 Identification of disease-related genes

Gene expression alterations associated with cervical cancer were examined using the publicly available GSE63514 PubMed ID: 26 056 290 (den Boon *et al.* 2015), and GSE9750 PubMed ID: 18 506 748 datasets retrieved from the Gene Expression Omnibus (GEO) repository (http://www.ncbi.nlm.nih.gov/geo/; accessed 4 July 2025). This dataset contains transcriptional profiles from cervical squamous cell carcinoma samples, along with matched noncancerous cervical tissues. Raw expression data were preprocessed and normalized prior to statistical evaluation. Differentially expressed genes (DEGs) were identified using a cutoff of adjusted *P* < .05 combined with (log_2_ fold change) > 1 (Deng *et al.* 2025). To identify candidate therapeutic targets of *P. notoginseng*, the list of DEGs was cross-referenced with the predicted molecular targets of its phytochemical constituents.

### 2.2 Development of compound datasets and pharmacokinetic profiling of *P. notoginseng*

Phytochemical compounds from *P. notoginseng* were retrieved from the TCM Systems Pharmacology Database and Analysis Platform (TCMSP; Chinese Academy of Sciences; Wei *et al.* 2024) and further verified using SwissADME (Lausanne, Switzerland; Table 1, available as supplementary data at *Bioinformatics Advances* online; Shang *et al.* 2023). Drug-likeness and pharmacokinetic properties were evaluated based on Lipinski’s rule of five (RO5) and key ADMET parameters, including molecular weight (MW; <500 g/mol), lipophilicity (LogP < 5), hydrogen bond donors (HBDs < 5), hydrogen bond acceptors ( HBAs < 7), intestinal absorption (>30%), blood-brain barrier (BBB) permeability (>1), and hepatotoxicity (Khare *et al.* 2023). Compounds satisfying these criteria were selected for subsequent analyses.

**Table 1:** Physiochemical and pharmacokinetic properties of *P. notoginseng* compounds.

Number	Compounds	Physiochemical properties				Pharmacokinetics properties		
		Molecular weight (g/mol) <500	Lipophilicity (LogP) <5	Hydrogen bond acceptors <7	Hydrogen bond donor <4	Intestinal absorption <30%(poorly absorbed)	BBB permeability >0.3 (Readily cross 0.3–0.1 moderatly absorbed), <-1 (poorly distributed)	Hepatoxicity Yes/No
1	()-beta-pinene	136.26	2.93	0	0	44.77	2.29	No
2	hepanal	204.39	4.36	0	0	53.83	2.17	No
3	(-)-alpha-cedrene	204.39	4.12	0	0	55.56	2.16	No
4	butylcyclobutane	112.24	3.45	0	0	47.59	2.15	No
5	1,2-dihydro-1,5,8-trimethylnaphthalene	172.29	4.12	0	0	47.69	2.13	No
6	alloaromadedrene	204.39	4.22	0	0	53.46	2.1	No
7	α-copaene	206.41	4.95	0	0	37.81	2.04	No
8	((1r)-1-methoxyethyl) benzene	136.21	2.01	1	0	42.09	1.89	No
9	butylated hydroxytoluene	220.39	4.85	1	1	40.02	1.8	No
10	wln: qr dg	128.56	2.23	1	1	60.44	1.75	No
11	hexenal	98.16	1.83	1	0	46.01	1.66	No
12	hypnon	120.16	1.57	1	0	48.19	1.54	No
13	hexanal	100.18	1.85	1	0	55.71	1.52	No
14	ptl	86.15	1.4	1	0	59.53	1.52	No
16	5-methylfurfural	110.12	1.13	2	0	43.92	1.48	No
17	2-acetylpyrrole	109.14	0.96	1	1	58.37	1.42	No
18	2,6-dimethyl-cyclohexanol	128.24	2.14	1	1	76.28	1.4	No
19	(5s)-5-ethyloxolan-2-one	114.16	1.18	2	0	75.69	1.4	No
20	1-hydroxycumene	136.21	1.81	1	1	59.97	1.36	No
21	mehq	124.15	1.55	2	1	43.98	1.33	No
22	Mandenol(Lenvatinib)	308.56	6.99	2	0	42	1.14	No
23	pel	122.18	1.55	1	1	44.03	1.13	No
24	ditertbutyl phthalate	278.38	3.4	4	0	43.67	1.13	No
25	dichloroaniline	162.02	2.41	1	2	44.51	1.08	No
26	stigmasterol	412.77	7.64	1	1	43.83	1	No
27	beta-sitosterol	414.79	8.08	1	1	36.91	0.99	No
28	linolenyl alcohol	264.5	6.02	1	1	42.79	0.97	No
29	hexanoic acid	116.18	1.81	2	1	73.08	0.93	No
30	zoomaric acid	254.46	5.92	2	1	35.78	0.88	No
31	10z, 13z-nonadecadienoic acid	294.53	6.85	2	1	40.98	0.87	No
32	isopulegone	154.23	0.99	2	0	55.39	0.81	No
33	oleic acid (Panaxadiol)	282.52	6.84	2	1	33.13	0.78	No
34	piceol	136.16	1.3	2	1	36.8	0.72	No
35	nsc692928	244.41	5.68	1	1	43.31	0.66	No
36	2-coumarate	164.17	1.64	3	2	53.6	0.28	No
37	diop(Daucosterol)	390.62	7.44	4	0	43.59	0.26	No

### 2.3 Identification of molecular targets associated with *P. notoginseng*

Cervical cancer-associated genes were retrieved from GeneCards, while potential targets of *P. notoginseng* phytochemicals were predicted using Swiss Target Prediction (Lausanne, Switzerland; Table 1, available as supplementary data at *Bioinformatics Advances* online; Shang *et al.* 2023). After removing redundant entries, overlapping genes between compound-related and disease-associated targets were identified using Venny 2.1 (National Center for Biotechnology, Madrid, Spain; Usmani Rana *et al.* 2024). These intersecting genes were considered core targets for subsequent mechanistic and pathway analyses.

### 2.4 PPI network for *P. notoginseng* targets in cervical cancer

A protein–protein interaction (PPI) network of *P. notoginsen* related targets in cervical cancer was constructed using the STRING database (v12.0; Heidelberg, Germany) with high-confidence interaction scores (≥0.9) to ensure robust, biologically reliable associations (Premkumar and Sajitha Lulu 2023, Table 1, available as supplementary data at *Bioinformatics Advances* online). The selected targets were uploaded to the database, and only experimentally validated and strongly predicted interactions were retained. The generated interaction network was then exported and visualized using Cytoscape (v3.10.2; San Diego, USA; Li *et al.* 2025b) to facilitate graphical interpretation. Topological analysis of the network was performed using the Network Analyzer tool integrated within Cytoscape. Key parameters, including degree centrality, betweenness centrality, and clustering coefficient, were calculated to evaluate node importance. Nodes with higher degree values were considered hub genes with potential regulatory significance. This approach enabled the identification of critical proteins involved in cervical cancer progression (last accessed 15 May 2025).

### 2.5 Gene ontology and pathway enrichment analysis of target genes related to cervical cancer and *P. notoginseng* compounds

Functional enrichment analysis of overlapping genes was performed to investigate their biological roles. Gene Ontology (GO) annotation was carried out using DAVID (v6.8; Bethesda, MD, USA; Sherman *et al.* 2022), while pathway enrichment analysis was conducted using the Kyoto Encyclopedia of Genes and Genomes (KEGG). A total of 410 high-confidence PPI genes (interaction score ≥ 0.9) were analyzed, and results were ranked by *P*-value, with the Benjamini-Hochberg correction applied to control the false discovery rate. Terms with adjusted *P* < .05 were considered significant. The top enriched GO categories and KEGG pathways were visualized using the SRplot platform (http://www.bioinformatics.com.cn); accessed 16 May 2025.

### 2.6 ML-based identification of key targets

To systematically identify therapeutic targets, the DEG sets derived from the GSE63514 and GSE9750 cervical cancer datasets were subjected to a multi-algorithm machine-learning framework. Prior to model construction, the DEG expression matrix was standardized (*z*-score normalization) to ensure comparability across features, and genes were used as input variables while sample classes (normal vs. cervical cancer) were defined as the response labels (Ayass *et al.* 2026). To avoid data leakage and ensure model generalize ability, the dataset was randomly divided into training (70%) and test (30%) sets using stratified sampling to preserve class distribution. All feature selection procedures were performed exclusively on the training dataset, and the test dataset was used only for independent evaluation.

Three complementary feature selection algorithms were independently applied to improve robustness and reduce model-specific bias. Least absolute shrinkage and selection operator (LASSO) regression was implemented using the glmnet package to perform penalized regression and shrink less informative coefficients toward zero, thereby selecting the most predictive gene subset (Dharmaratne *et al.* 2025). Support vector machine recursive feature elimination (SVM-RFE), implemented through the caret and e1071 packages, was used to iteratively eliminate low-weight features based on classification performance to identify an optimal gene signature. In parallel, Random forest (RF) analysis was conducted using the RF package to rank genes according to their importance scores derived from ensemble decision trees (Liu *et al.* 2021a).

To further prevent overfitting and assess model robustness, 5-fold cross-validation was applied on the training set during model development. Genes consistently identified across all three ML models were defined as robust hub targets associated with cervical cancer and the pharmacological effects of *P. notoginseng*.

To evaluate their diagnostic performance, these intersecting hub genes were incorporated into a multivariate logistic regression (LR) model (Huang *et al.* 2022), Model performance was assessed using receiver operating characteristic (ROC) curve analysis, with evaluation conducted on the training set, cross-validation folds, and independent test set. This multi-level validation strategy ensured reliable estimation of model performance and minimized the risk of overfitting.

### 2.7 Identification of key hub genes targeted by *P. notoginseng* compounds in cervical cancer

A topological analysis of the primary PPI network was conducted to identify the most influential nodes. Degree and shortest-path distance were used as key centrality measures to identify genes with high connectivity and regulatory importance. Overlapping genes across these measures were identified using the Venny tool and designated as hub genes (Yan *et al.* 2023). Final hub gene selection and network evaluation were performed using the cytoHubba plugin in Cytoscape v3.10.2, focusing on *P. notoginseng* targets relevant to cervical cancer (Aziz *et al.* 2023).

### 2.8 Molecular docking analysis of *P. notoginseng* derived compounds with hub proteins associated with cervical cancer

Molecular docking was performed to validate network pharmacology findings by evaluating interactions between *P. notoginseng* bioactive compounds and key cervical cancer-associated hub proteins (TNF-α, IL-6, and SRC). Ligand structures were retrieved from PubChem (Bethesda, MD, USA) and optimized using UCSF Chimera (v1.17.3; Pettersen *et al.* 2021), while target protein crystal structures (PDB IDs: 4V46, 7PHS, and 2JYQ) were obtained from the RCSB Protein Data Bank and prepared by removing water molecules and co-crystallized ligands using PyMOL (v2.4.0). Docking simulations were conducted using the CB-Dock2 platform (Liu *et al.* 2022), which integrates cavity detection (CurPocket) and template-guided docking (FitDock) to predict binding poses and affinities. Binding interactions were assessed based on docking scores (≤−6.0 kcal/mol considered significant), hydrogen bond distance (≤3.5 Å), and hydrophobic contacts (≤5.0 Å). The best-ranked ligand–protein complexes were selected based on binding energy and interaction stability for further analysis.

### 2.9 MD simulation of *P. notoginseng* compounds with cervical cancer targets

To investigate the conformational stability, flexibility, and interaction dynamics of Lenvatinib, and *P. notoginseng* compounds (Ginsenoside Re, Panaxadiol, Stigmasterol, Daucosterol) with cervical cancer-associated protein targets (IL6, TNF-α, SRC), MDs simulations were performed using the Desmond module of Schrödinger Suite 2021–4 (Wang *et al.* 2024). All protein–ligand complexes were prepared using the Protein Preparation Wizard, parameterized with the OPLS4 force field, and solvated in a cubic box using the SPC water model (Mfeka *et al.* 2025). System neutrality was achieved by adding counterions, and the simulations were run for 200 ns under NPT ensemble conditions (310 K, 1 atm) using the Berendsen thermostat and barostat.

RMSD was calculated to monitor the overall structural deviation of protein–ligand complexes over time. The RMSD is defined by:


(1) $${\mathrm{RMSD}}_{X}=\sqrt{1/N\sum_{i=1}^{n}(r'i(tx)){-ri(\mathrm{tref}))}^{2}}$$

where $\vec{\text{ri}}(tx)$ is the atomic position at time *txt*, $\vec{\text{ri}}(t\text{ref})$ is the reference position, and *N* is the number of backbone atoms. Low and stable RMSD values throughout the trajectory indicate global structural stability of the complex (Ormeño and General 2024).

RMSF was employed to assess residue-level flexibility over the 200 ns simulation:


(2) $${\mathrm{RMSF}}_{i}\;=\;\sqrt{\sum <(r'i(t)){-ri(\mathrm{tref}))}^{2}>}$$

where ⟨r i⟩ is the time-averaged position of atom *i*. Higher RMSF values correspond to greater flexibility, often localized in loop or terminal regions, while lower values denote rigid structural cores (Song *et al.* 2024).

The time-dependent evolution of the secondary structural elements (SSEs) was assessed using Desmond’s Timeline analysis tool. Both per-residue secondary structure percentages and time-resolved SSE maps were generated for each complex (Shukla *et al.* 2017). The presence and persistence of α-helices, β-strands, and loops were monitored across the trajectory, providing insight into local folding/unfolding events and conformational transitions. The stability of secondary structures indicates that protein integrity is preserved upon ligand binding.

Protein–ligand interactions were evaluated using the Simulation Interaction Diagram tool in Desmond, focusing on hydrogen bonds (donor acceptor ≤2.5 Å, angle ≥120°), hydrophobic contacts (3.6–4.5 Å), ionic interactions (≤3.7 Å), and water bridges (Martis *et al.* 2023). Interaction fractions quantified the proportion of simulation time during which residues remained in contact with ligands, with values >1 indicating simultaneous multiple interactions (Frey *et al.* 2022). This analysis enabled the identification of key stabilizing residues crucial for complex stability and biological activity.

To further characterize collective motions and assess trajectory convergence, principal component analysis (PCA) was performed on the MD trajectories (Roccatano 2025). Covariance matrices of atomic positional fluctuations were constructed and diagonalized to extract principal components representing dominant conformational motions (Rahimi *et al.* 2023). The first two principal components (PC1 and PC2) were used to project the trajectory and evaluate conformational space sampling and system stability.

Additionally, dynamic cross-correlation matrix (DCCM) analysis was conducted to examine correlated and anti-correlated motions between residue pairs throughout the simulation. Cross-correlation coefficients were calculated based on atomic displacement vectors, where positive values indicate coordinated motions and negative values represent anti-correlated movements (dos Santos Nascimento *et al.* 2022). This analysis provided insights into intra-protein communication and the influence of ligand binding on residue-level dynamics.

### 2.10 Statistical analysis

All statistical analyses were performed in R (v4.4.3). Differential expression analysis of the GSE63514 and GSE9750 cervical cancer datasets was conducted using the limma package. Functional enrichment analyses (GO and KEGG) were performed with clusterProfiler and visualized using ggplot2. ML-based feature selection was performed using LASSO (glmnet), SVM-RFE (caret, e1071), and RF (randomForest), while the diagnostic performance of hub genes was assessed via ROC analysis with pROC. Single-cell RNA-seq data, where applicable, were analyzed using Seurat. Statistical significance was set at *P* < .05.

## 3 Results

### 3.1 Differential gene expression and pathway enrichment in cervical cancer

**Figure 2:**
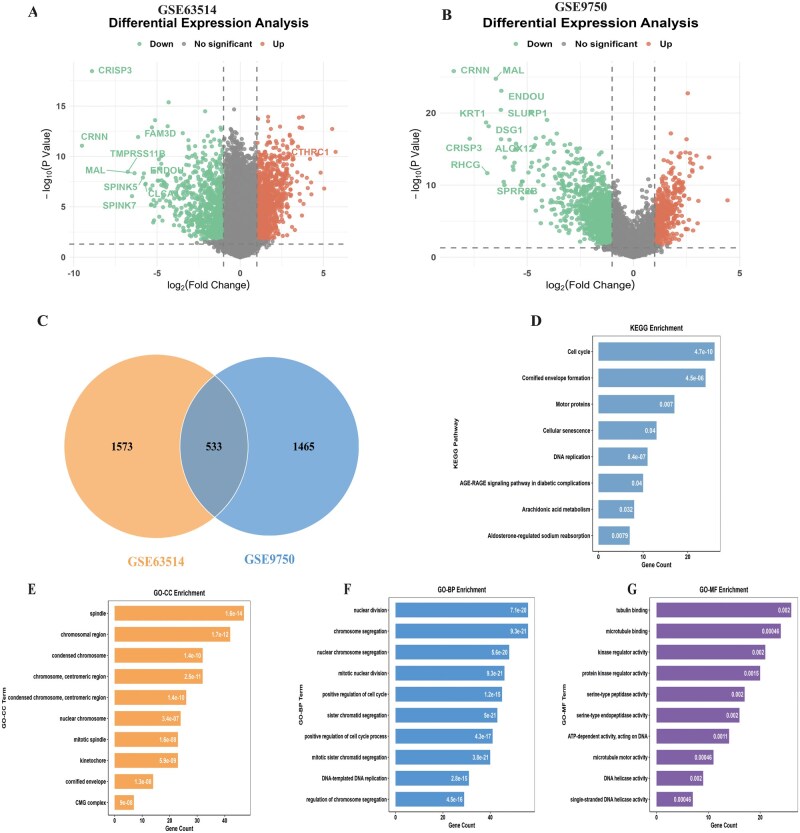
Differential expression and functional enrichment analysis of cervical cancer datasets. (A and B) Volcano plots showing DEGs in the GSE63514 (A) and GSE9750 (B) datasets. (C) Venn diagram illustrating the overlap of DEGs between the two datasets. (D) KEGG pathway enrichment analysis of the shared DEGs. (E) GO enrichment analysis for CC. (F) GO enrichment analysis for BP. (G) GO enrichment analysis for MF. All enrichment analyses were performed using an adjusted *P*-value < .05 as the significance threshold.

To comprehensively characterize transcriptomic alterations in cervical cancer, differential expression analysis was performed on the GEO datasets GSE63514 and GSE9750 using the criteria log_2_ fold change (log_2_FC) ≥ 1 and adjusted *P*-value < .05. As shown in the volcano plots (Fig. 2a and b), a total of 2106 DEGs were identified in GSE63514 and 1998 DEGs in GSE9750, including significantly upregulated and downregulated genes, indicating substantial transcriptional dysregulation between normal and tumor samples. We perform an intersection analysis (Fig. 2c), which reveals 533 overlapping DEGs, along with 1573 genes unique to GSE63514 and 1,465 genes unique to GSE9750. These shared genes were considered high-confidence candidates for subsequent functional analysis. Functional enrichment analysis of overlapping DEGs revealed significant enrichment in key oncogenic pathways. KEGG pathway analysis (Fig. 2d) showed enrichment in pathways such as cell cycle, DNA replication, cellular senescence, and AGE-RAGE signaling, which are closely associated with tumor proliferation, genomic instability, and cancer progression (Fig. 1, available as supplementary data at *Bioinformatics Advances* online).

Consistent with KEGG results, GO enrichment analysis revealed strong associations with mitotic regulation. In the cellular component (CC) category (Fig. 2e), enriched terms included spindle, chromosomal region, condensed chromosome, and kinetochore, indicating alterations in chromosome organization and segregation. In the biological process (BP) category (Fig. 2f), DEGs were significantly enriched in nuclear division, chromosome segregation, mitotic nuclear division, and DNA replication, reflecting dysregulation of cell cycle progression. In the molecular function (MF) category (Fig. 2g), enrichment was observed in microtubule binding, tubulin binding, kinase regulator activity, and ATP-dependent activity acting on DNA, suggesting disruptions in cytoskeletal dynamics and enzymatic regulation.

These findings demonstrate that cervical cancer-associated transcriptomic alterations are predominantly driven by dysregulation of cell cycle progression, mitotic machinery, and DNA replication, with consistent patterns observed across independent datasets. These results provide a reliable transcriptomic basis for subsequent integrative analyses exploring the multi-target mechanisms of *P. notoginseng* in cervical cancer.

### 3.2 Transcriptomic landscape of cervical cancer reveals mitotic deregulation and immune–metabolic crosstalk

**Figure 3:**
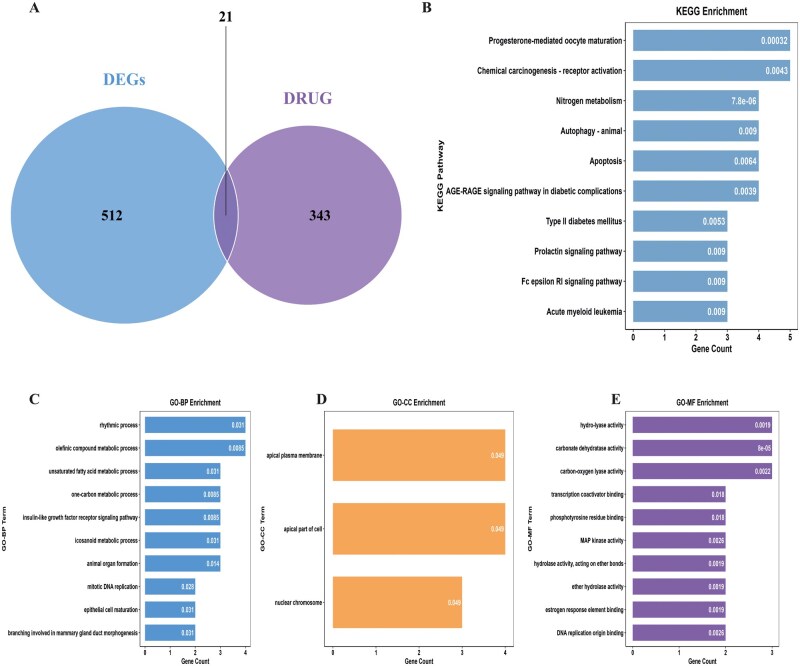
Integration of transcriptomic and drug target analysis with functional enrichment in cervical cancer. (A) Venn diagram showing the overlap between DEGs and predicted drug targets. (B) KEGG pathway enrichment analysis of the overlapping genes. (C) GO enrichment analysis for BP. (D) GO enrichment analysis for CC. (E) GO enrichment analysis for MF.

A comparative analysis integrating DEGs and predicted drug targets (Fig. 3a) identified 21 overlapping genes among 512 DEGs and 343 drug-associated targets, representing potential therapeutic candidates for cervical cancer. KEGG pathway enrichment analysis (Fig. 3b) of the overlapping genes revealed significant enrichment in pathways such as progesterone-mediated oocyte maturation, chemical carcinogenesis–receptor activation, nitrogen metabolism, autophagy, apoptosis, AGE-RAGE signaling, and prolactin signaling, indicating involvement in cellular stress responses, metabolic regulation, and tumor-related signaling processes. GO enrichment analysis further characterized the functional roles of these genes. In the BP category (Fig. 3c), enriched terms included rhythmic process, metabolic processes (olefinic compound, unsaturated fatty acid, and one-carbon metabolism), insulin-like growth factor receptor signaling, and mitotic DNA replication, suggesting roles in metabolic regulation and cellular proliferation. In the CC category (Fig. 3d), enrichment was observed in the apical plasma membrane, the apical part of the cell, and the nuclear chromosome, indicating localization associated with membrane structures and chromosomal regions. In the MF category (Fig. 3e), significant terms included hydrolase activity, carbonate dehydratase activity, transcription coactivator binding, phosphotyrosine residue binding, Mitogen-Activated Protein (MAP) kinase activity, and DNA replication origin binding, reflecting enzymatic activity and regulatory functions in signaling and DNA-related processes.

Overall, these results highlight a subset of candidate genes linking cervical cancer transcriptomic alterations to potential drug targets, primarily associated with metabolic pathways, signaling regulation, and cellular stress responses, providing a basis for further therapeutic investigation of *P. notoginseng*.

### 3.3 ML-based identification of hub genes in cervical cancer

**Figure 4:**
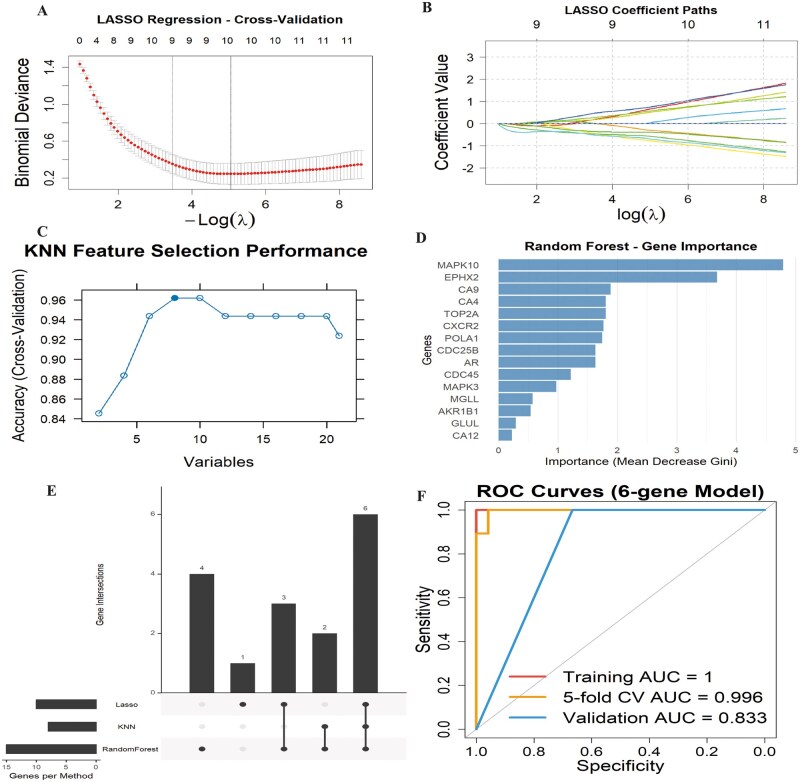
Machine learning-based identification of hub genes in cervical cancer. (A) LASSO regression cross-validation curve showing the selection of the optimal penalty parameter (λ) based on minimum binomial deviance. (B) LASSO coefficient profiles of candidate genes across varying λ values, illustrating coefficient shrinkage with increasing regularization. (C) KNN feature selection performance showing cross-validation accuracy across different numbers of variables. (D) RF analysis ranking genes based on importance (Mean Decrease Gini). (E) Overlap of candidate genes identified by LASSO, KNN, and RF methods. (F) ROC curves of the logistic regression model showing performance in the training set (AUC = 1.0), 5-fold cross-validation (AUC = 0.996), and validation set (AUC = 0.833).

To refine the identification of key molecular drivers in cervical cancer, multiple ML approaches by using candidate genes derived from the integrated differential expression analysis of GSE63514 and GSE9750. LASSO regression with cross-validation (Fig. 4a) identified the optimal penalty parameter (λ) that minimized binomial deviance, enabling selection of the most informative features. The corresponding coefficient profile plot (Fig. 4b) illustrates the progressive shrinkage of gene coefficients toward zero with increasing regularization, indicating effective feature reduction. K-nearest neighbor (KNN) feature selection analysis (Fig. 4c) showed that model performance improved with increasing variables, achieving optimal cross-validation accuracy at approximately 9–11 variables, after which performance plateaued, suggesting an optimal feature subset within this range. RF analysis (Fig. 4d) ranked genes based on importance (Mean Decrease Gini), with MAPK10, EPHX2, CA9, CA4, and TOP2A identified as the most influential predictors. Additional important genes included CXCR2, POLA1, CDC25B, AR, and CDC45, highlighting their potential roles in cervical cancer biology. Integration of candidate genes across the three ML methods (Fig. 4e) revealed a subset of overlapping genes shared between LASSO, KNN, and RF, representing high-confidence biomarkers derived from multi-method consensus. To evaluate predictive performance, a LR model based on six selected genes was constructed. The ROC curves (Fig. 4f) demonstrated strong diagnostic performance, with AUCs of 1.0 for the training set, 0.996 for 5-fold cross-validation, and 0.833 for the validation set, indicating high accuracy with reasonable generalization.

These findings demonstrate that integrating transcriptomic data from multiple datasets (GSE63514 and GSE9750) with ML approaches provides a robust strategy for identifying reliable diagnostic biomarkers in cervical cancer.

### 3.4 Physicochemical and pharmacokinetic profiling of *P. notoginseng* compounds

A comprehensive in silico screening of 37 phytochemicals derived from *P. notoginseng* was performed using data from the TCMSP and SwissADME databases to evaluate key drug-likeness parameters. These included MW, lipophilicity (LogP), HBAs, HBDs, intestinal absorption, BBB permeability, and hepatotoxicity (Ma *et al.* 2021). The majority of the compounds satisfied Lipinski’s RO5 (Nhlapho *et al.* 2024), indicating acceptable oral bioavailability and drug-likeness (Table 1). Furthermore, none of the compounds were predicted to exhibit hepatotoxicity, underscoring their safety profiles. Among these, eight compounds, Sorafenib, Sunitinib, Lenvatinib, Stigmasterol, Daucosterol, Ginsenoside Re, Panaxadiol, D-Mannitol, β-Sitosterol, and Ginsenoside Rg3, were selected for downstream docking and MDs simulation studies based on a multi-parameter rationale. These compounds either demonstrated high intestinal absorption (>40%) and moderate-to-high BBB permeability (0.66–1.0), or were known from literature for their anticancer, anti-inflammatory, and immune-modulatory effects relevant to cervical cancer pathophysiology. For instance, Sorafenib, Sunitinib, and Lenvatinib, clinically approved tyrosine kinase inhibitors (TKIs), served as a positive control due to their strong binding affinity toward oncogenic kinases such as SRC and TNF-α. Ginsenoside Re and Ginsenoside Re3 were prioritized for their documented inhibition of IL6 signaling, while stigmasterol and β-sitosterol were retained despite exceeding standard MW and LogP thresholds due to their potent antiproliferative and pro-apoptotic activity in hormone-responsive cancers (Kim *et al.* 2025, Table 1). Daucosterol, Panaxadiol, and D-Mannitol were included based on their predicted high docking scores, structural compatibility with inflammatory targets (IL6), and stable RMSD profiles observed during preliminary MD simulations.

Together, these pharmacokinetic assessments provided a strong basis for selecting bioactive compounds with optimal ADME characteristics and therapeutic relevance, supporting their inclusion in protein–ligand interaction studies targeting key inflammatory and oncogenic mediators in cervical cancer.

### 3.5 Identification of overlapping targets and functional enrichment of *P. notoginseng* against cervical cancer

**Figure 5:**
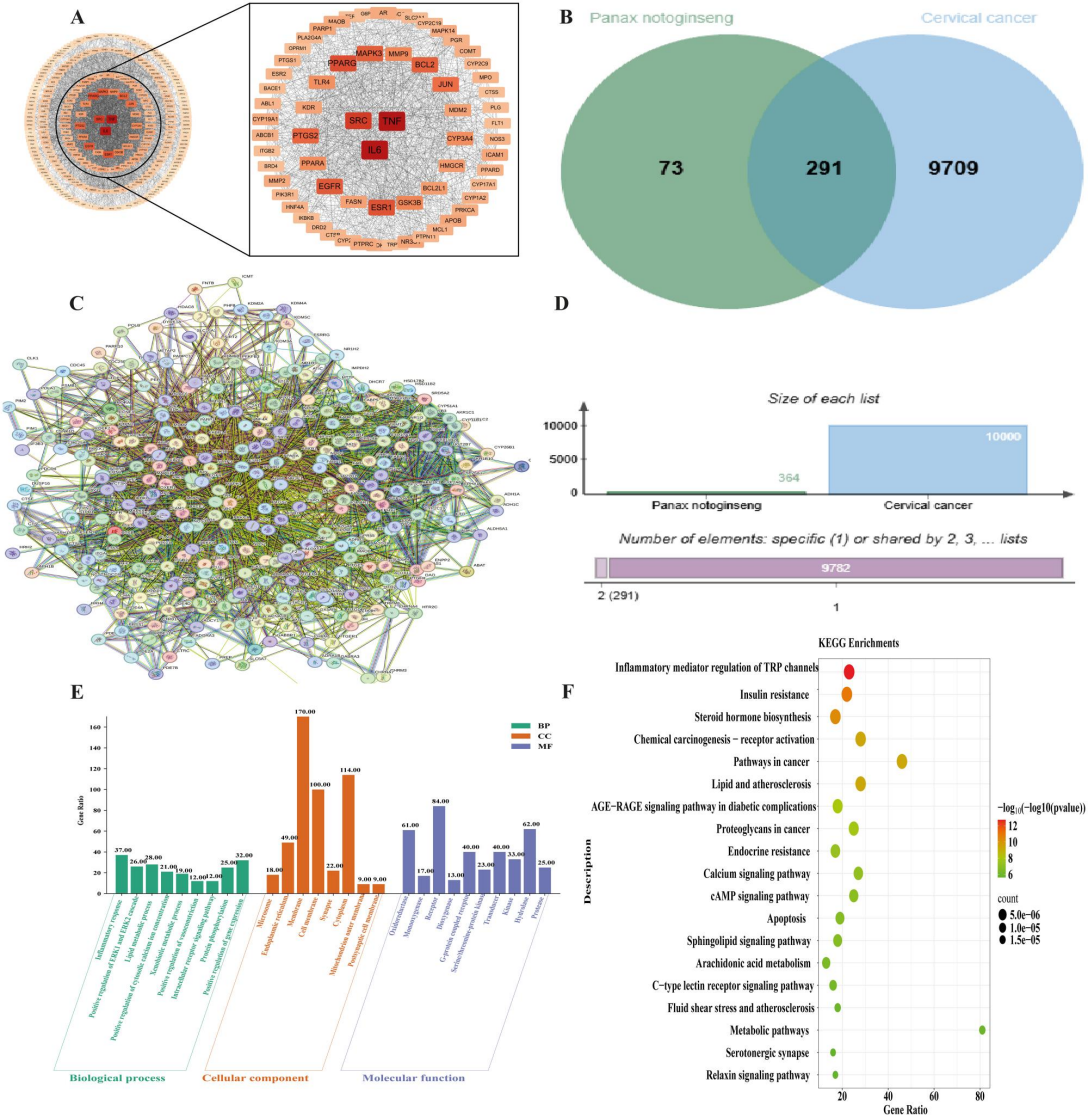
Identification of overlapping targets and functional enrichment analysis of *P. notoginseng* in cervical cancer. (A) Compound disease interaction network showing hub genes (TNF-α, SRC, IL6) and associated targets. (B) Venn diagram illustrating the overlap between *P. notoginseng* related targets and cervical cancer-related targets. (C) PPI network of the overlapping targets constructed using the STRING database. (D) Hub gene analysis identifying key targets involved in tumor-promoting inflammation and proliferative signaling. (E) GO enrichment results, including BP, CC, and MF categories. (F) KEGG pathway enrichment highlighting cancer-related and metabolism-associated pathways.

To elucidate the therapeutic potential of *P. notoginseng* in cervical cancer, a network pharmacology approach was employed. A total of 364 *P. notoginseng*-associated targets and 10 000 cervical cancer-related genes were retrieved, yielding 291 overlapping targets with potential pharmacological relevance (Fig. 5b–d). The compound-disease interaction network demonstrated a highly interconnected topology, where hub analysis identified TNF-α, SRC, and IL6 (Bent *et al.* 2021) as central nodes (Fig. 3a). These core regulators are essential mediators of tumor-promoting inflammation, angiogenesis, immune evasion, and proliferative signaling in cervical cancer. Additional critical targets, including MAPK3, EGFR, BCL2, and ESR1 (Liu *et al.* 2023), were also highlighted, supporting the multi-faceted regulatory role of *P. notoginseng*. PPI analysis revealed a densely clustered and highly connected network, indicating that these overlapping targets function cooperatively within key oncogenic pathways (Fig. 5c). Functional enrichment analyses were conducted to further define the molecular basis of these interactions. KEGG pathway analysis revealed significant enrichment in tumor and metabolism-associated pathways, including inflammatory mediator regulation of TRP channels, insulin resistance, steroid hormone biosynthesis, chemical carcinogenesis, proteoglycans in cancer, and endocrine resistance (Fig. 5f). These findings suggest that *P. notoginseng* may disrupt multiple hallmarks of cervical cancer, including metabolic reprogramming, hormone-driven carcinogenesis, and receptor-mediated signaling. GO enrichment further reinforced these observations. GO–BP terms highlighted involvement in inflammatory responses, regulation of the ERK1/2 cascade, lipid metabolism, and calcium ion signaling, processes essential to cervical tumor growth and therapeutic resistance. GO–CC) analysis indicated localization to the plasma membrane, endoplasmic reticulum, synaptic membranes, and vesicular compartments, reflecting regulation of protein processing, intercellular communication, and ER stress-related apoptosis (Fig. 5e). GO–MF analysis underscored enrichment in mono-oxygenase activity, G-protein coupled receptor (GPCR) activity, and serine/threonine kinase activity, which are directly linked to the inflammatory (TNF-α, IL6) and oncogenic (SRC, MAPK) signaling axes.

Together, these findings reveal that *P. notoginseng* exerts its therapeutic effects through a multi-target, multi-pathway regulatory mechanism. By simultaneously modulating TNF-α, SRC, and IL6 signaling axes alongside other oncogenic and metabolic pathways, *P. notoginseng* demonstrates strong potential to attenuate chronic inflammation, inhibit tumor-promoting signaling, and restore immune balance in cervical cancer.

### 3.6 Comparative docking analysis of IL6 with *P. notoginseng* derived compounds

**Figure 6:**
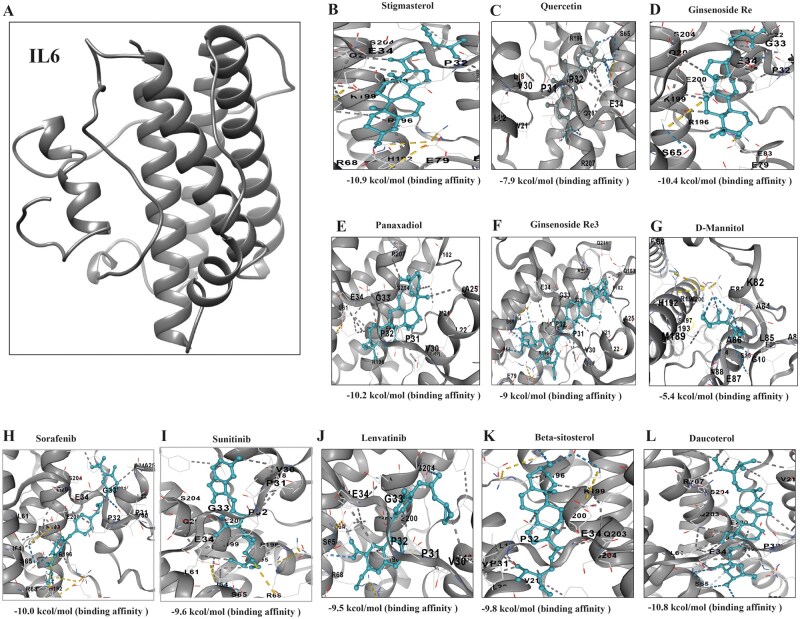
Molecular docking analysis of IL6 with bioactive compounds from *P. notoginseng* and clinically used tyrosine kinase inhibitors. (A) 3D ribbon structure of the human IL6 protein used for docking analysis. Docked binding poses of IL6 with (B) Stigmasterol, (C) Quercetin, (D) Ginsenoside Re, (E) Panaxadiol, (F) Ginsenoside Re3, (G) D-Mannitol, (H) Sorafenib, (I) Sunitinib, (J) Lenvatinib, (K) β-sitosterol, and (L) Daucosterol. Binding affinities are presented in kcal/mol.

To investigate the IL6-targeting potential of both natural compounds and clinically used therapies, molecular docking analysis was performed using selected bioactive constituents from *P. notoginseng* alongside three clinically approved TKIs, Lenvatinib, Sorafenib, and Sunitinib (Fig. 6a–l). In Fig. 6, Lenvatinib exhibited a binding affinity of −9.5 kcal/mol, interacting with key residues, including E34, Q203, and P31, at the IL6 binding interface (Fig. 6j). Similarly, Sorafenib and Sunitinib demonstrated binding affinities of −10.0 kcal/mol and −9.6 kcal/mol, respectively, forming interactions with functionally relevant residues in the IL6 active site (Fig. 6h and i). Among the *P. notoginseng* compounds, several showed relatively strong predicted binding affinities. Stigmasterol (−10.9 kcal/mol; Fig. 6b), Daucosterol (−10.8 kcal/mol; Fig. 6l), Ginsenoside Re (−10.4 kcal/mol; Fig. 6d), and Panaxadiol (−10.2 kcal/mol; Fig. 6e) demonstrated comparable docking scores and formed multiple hydrogen bonding interactions with key IL6 residues, including E34, R68, S65, and Q203. β-sitosterol (−9.8 kcal/mol; Fig. 6k) and Ginsenoside Re3 (−9.0 kcal/mol; Fig. 6f) showed moderate binding affinity and stable interaction profiles.

In contrast, D-Mannitol (−5.4 kcal/mol; Fig. 6g) and Quercetin (−7.9 kcal/mol; Fig. 6c) exhibited comparatively weaker binding affinities and fewer stabilizing interactions within the binding pocket. These findings suggest that several *P. notoginseng* compounds exhibit predicted binding interactions with IL6, with docking scores comparable to those of selected TKIs. However, further experimental validation is required to confirm their functional relevance in modulating IL6-mediated signaling pathways.

### 3.7 Comparative docking analysis of *P. notoginseng* derived compounds and TKIs with SRC kinase

**Figure 7:**
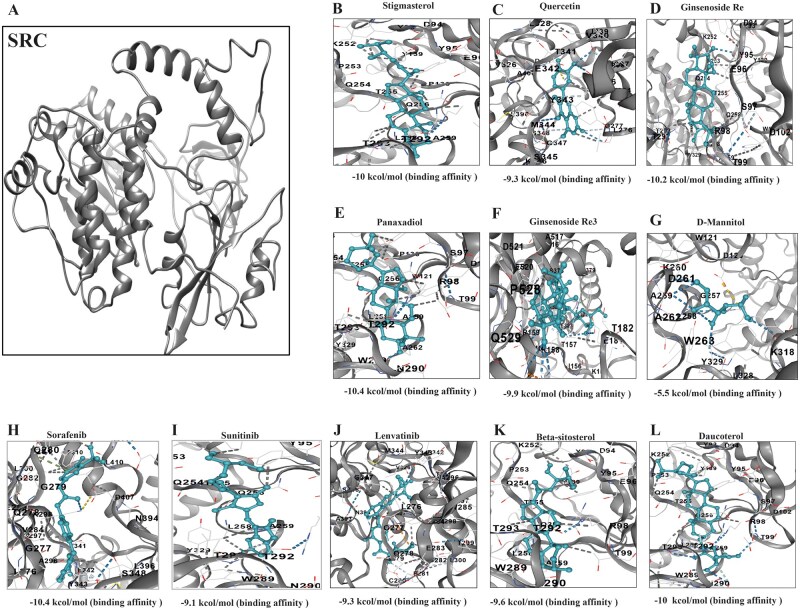
Comparative molecular docking analysis of SRC kinase with bioactive compounds from *P. notoginseng* and clinically relevant tyrosine kinase inhibitors. (A) Ribbon representation of the human SRC kinase structure used for docking analysis. Docked binding conformations of SRC with (B) Stigmasterol, (C) Quercetin, (D) Ginsenoside Re, (E) Panaxadiol, (F) Ginsenoside Re3, (G) D-Mannitol, (H) Sorafenib, (I) Sunitinib, (J) Lenvatinib, (K) β-sitosterol, and (L) Daucosterol. Binding affinities are indicated in kcal/mol.

To evaluate the potential interaction of *P. notoginseng* compounds with the oncogenic SRC kinase, molecular docking was performed and compared with three clinically used TKIs, Lenvatinib, Sorafenib, and Sunitinib (Fig. 7a–l). As shown in Fig. 7, Lenvatinib exhibited a binding affinity of −9.3 kcal/mol, interacting with key residues within the SRC active site, including Q254, D261, and K252 (Fig. 7j). Similarly, Sorafenib and Sunitinib demonstrated binding affinities of −10.4 kcal/mol and −9.1 kcal/mol, respectively, forming interactions with functionally relevant residues in the ATP-binding pocket of SRC (Fig. 7h and i). Among the *P. notoginseng* compounds, several molecules showed relatively strong predicted binding affinities. Panaxadiol (−10.4 kcal/mol; Fig. 7e), Ginsenoside Re (−10.2 kcal/mol; Fig. 7d), Stigmasterol (−10.0 kcal/mol; Fig. 7b), and Daucosterol (−10.0 kcal/mol; Fig. 7l) exhibited comparable docking scores and formed multiple interactions with key SRC residues such as T293, W289, and D261. Ginsenoside Re3 (−9.9 kcal/mol; Fig. 7f) and β-sitosterol (−9.6 kcal/mol; Fig. 7k) showed moderate binding affinity with stable interaction profiles, while Quercetin (−9.3 kcal/mol; Fig. 7c) demonstrated comparable binding to Lenvatinib. In contrast, D-Mannitol (−5.5 kcal/mol; Fig. 7g) exhibited relatively weak binding affinity and limited interactions within the binding pocket, supporting the specificity of the docking analysis. Detailed binding energies are provided in Table 2, available as supplementary data at *Bioinformatics Advances* online.

Overall, these results suggest that several *P. notoginseng* compounds exhibit predicted binding interactions with SRC kinase, with docking scores comparable to those of clinically used TKIs.

### 3.8 TNF-α binding affinity comparison between *P. notoginseng* derived phytochemicals and lenvatinib

**Figure 8:**
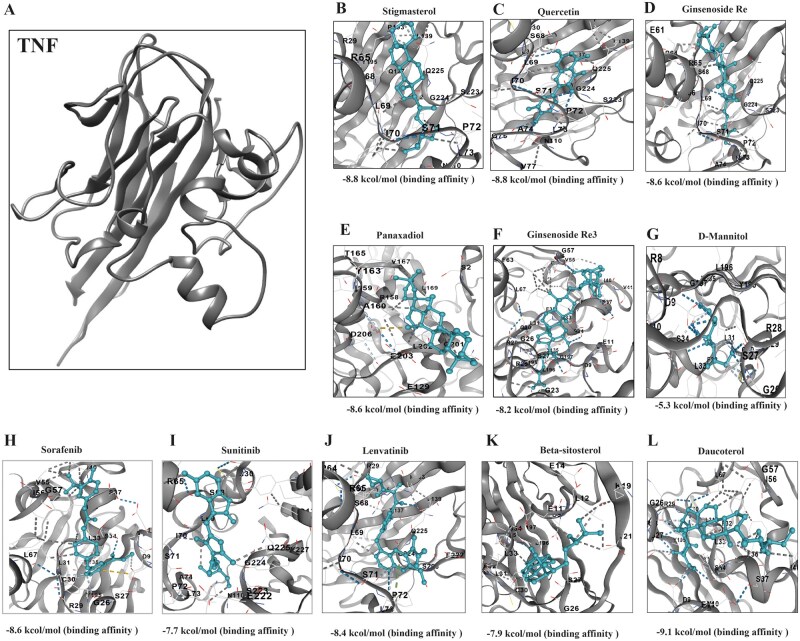
Molecular docking analysis of TNF with bioactive compounds from *P. notoginseng* and reference drugs. (A) Three-dimensional structure of TNF. Binding interactions of TNF with (B) stigmasterol, (C) quercetin, (D) ginsenoside Re, (E) panaxadiol, (F) ginsenoside Re3, (G) D-mannitol, (H) sorafenib, (I) sunitinib, (J) lenvatinib, (K) β-sitosterol, and (L) daucosterol. Binding affinities (kcal/mol) are indicated for each complex. Hydrogen bonds are represented by dashed lines, while hydrophobic interactions are shown as non-bonded contacts.

Molecular docking was performed to evaluate the binding interactions between tumor necrosis factor-α (TNF-α) and selected phytochemicals from *P. notoginseng*, with comparisons to clinically used reference TKIs. As shown in (Fig. 8), all tested compounds successfully occupied the active binding pocket of TNF-α, engaging key stabilizing residues including Arg, Leu, Gly, Pro, and Ser. Among the phytochemicals, Stigmasterol and Quercetin exhibited strong binding affinities (−8.8 kcal/mol; Fig. 8b and c), forming multiple hydrogen bonds and hydrophobic interactions with key residues such as Arg29, Leu63, and Gly224. Ginsenoside Re and Panaxadiol also showed comparable binding energies (−8.6 kcal/mol; Fig. 8d and e), stabilized through combined hydrogen bonding interactions. Ginsenoside Re3 demonstrated moderate binding affinity (−8.2 kcal/mol; Fig. 8f), whereas D-mannitol showed weak interaction (−5.3 kcal/mol; Fig. 8g), indicating limited binding stability. Compared with standard anticancer TKIs, sorafenib showed a binding affinity of −8.6 kcal/mol (Fig. 8h), sunitinib −7.7 kcal/mol (Fig. 8i), and lenvatinib −8.4 kcal/mol (Fig. 8j), all forming stable interactions within the TNF-α binding pocket. Notably, Daucosterol exhibited the highest binding affinity (−9.1 kcal/mol; Fig. 8l), indicating a stable and energetically comparable interaction, while Beta-sitosterol showed moderate binding (−7.9 kcal/mol; Fig. 8k; binding affinity energies are shown in Table 2, available as supplementary data at *Bioinformatics Advances* online).

Overall, the docking results indicate that key *P. notoginseng* compounds daucosterol, stigmasterol, quercetin, ginsenoside Re, and panaxadiol exhibit binding affinities comparable to or higher than standard therapeutic agents, suggesting their potential to effectively occupy the TNF-α active site and modulate TNF-α mediated inflammatory signaling in cancer.

### 3.9 MDs simulation analysis of IL-6, SRC, and TNF complexes

**Figure 9:**
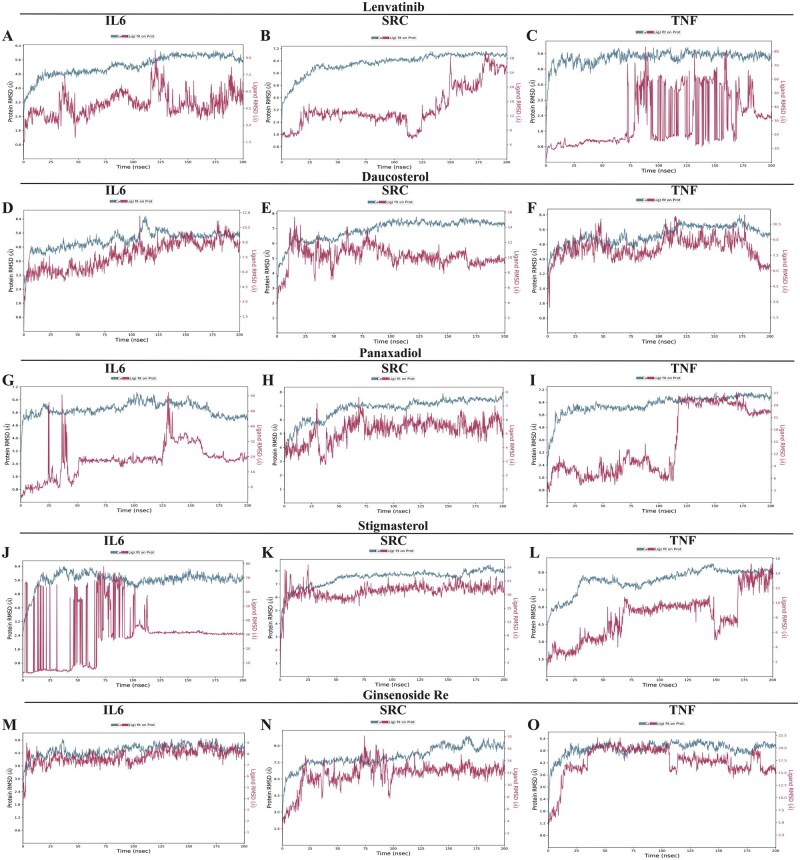
MD simulation analysis of protein–ligand complexes. RMSD profiles of IL-6, SRC, and TNF in complex with *P. notoginseng* compounds and the reference drug Lenvatinib over 200 ns simulations. (A–C) Lenvatinib complexes with IL-6, SRC, and TNF. (D–F) Daucosterol complexes with IL-6, SRC, and TNF. (G–I) Panaxadiol complexes with IL-6, SRC, and TNF. (J–L) Stigmasterol complexes with IL-6, SRC, and TNF. (M–O) Ginsenoside Re complexes with IL-6, SRC, and TNF.

We further evaluate the stability and dynamic behavior of the docked complexes. 200 ns MDs simulations were performed for IL-6, SRC, and TNF proteins in complex with selected *P. notoginseng* compounds and the reference drug Lenvatinib. The root-mean-square deviation (RMSD) of protein backbone atoms and ligand positions was analyzed to assess structural stability over time (Fig. 9). The Lenvatinib-bound complexes (Fig. 9a–c) exhibited generally stable RMSD profiles for IL-6 and SRC, reaching equilibrium after initial fluctuations, whereas TNF showed greater variability, indicating moderate conformational flexibility. Compared with *P. notoginseng* compounds, Daucosterol-bound systems (Fig. 9d–f) showed consistent, stable RMSD trajectories across all three targets, suggesting comparable binding stability. Similarly, Panaxadiol complexes (Fig. 9g–i) showed gradual stabilization, with IL-6 and SRC maintaining relatively steady RMSD values, while TNF exhibited minor fluctuations before equilibrium. Stigmasterol-bound complexes (Fig. 9j–l) displayed moderate stability, with SRC and TNF maintaining stable conformations, whereas IL-6 showed noticeable fluctuations during the early phase before stabilizing. Ginsenoside Re-bound systems (Fig. 9m–o) exhibited stable RMSD profiles, particularly for IL-6 and TNF, while SRC showed moderate but controlled deviations, indicating acceptable conformational stability.

MD simulation results indicate that Daucosterol, Panaxadiol, Stigmasterol, and Ginsenoside Re, form a stable complex with IL-6, SRC, and TNF, maintaining structural integrity throughout the simulation. In comparison, the reference drug, Lenvatinib, showed greater fluctuations in TNF, suggesting lower stability at this target. All systems exhibited an initial equilibration phase within the first ∼20 ns, after which RMSD values stabilized; therefore, structural stability was evaluated based on the post-equilibration trajectory.

### 3.10 RMSF-based evaluation of residue-level dynamics in protein–ligand complexes

**Figure 10:**
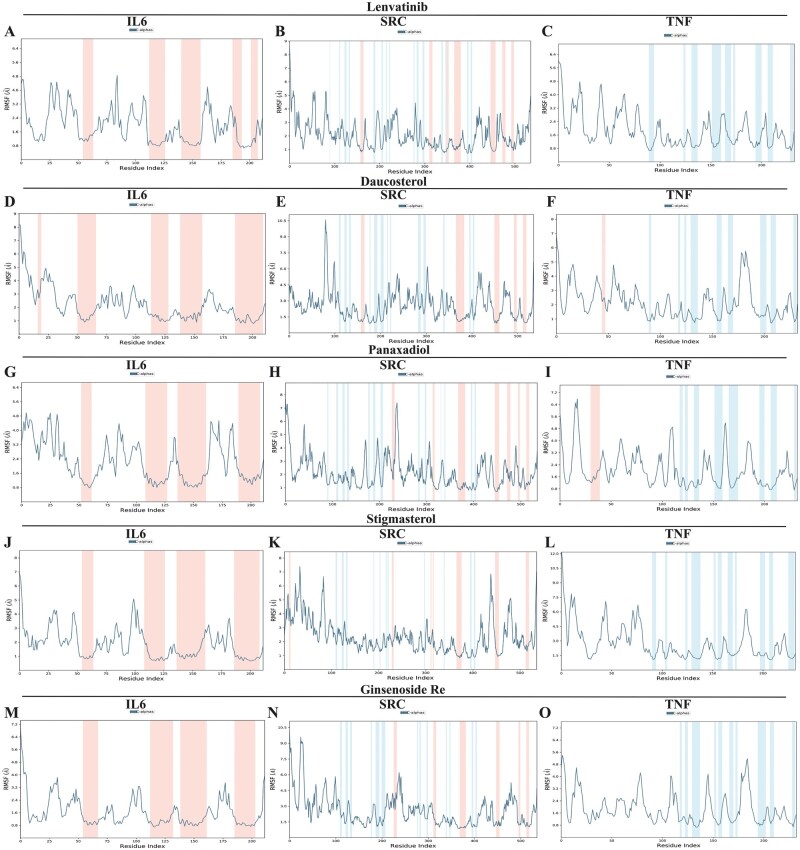
RMSF analysis of protein–ligand complexes. RMSF profiles of protein backbone atoms for IL-6, SRC, and TNF in complex with *P. notoginseng* compounds and the reference drug Lenvatinib over 200 ns molecular dynamics simulations. (A–C) Lenvatinib complexes with IL-6 (A), SRC (B), and TNF (C). (D–F) Daucosterol complexes with IL-6 (D), SRC (E), and TNF (F). (G–I) Panaxadiol complexes with IL-6 (G), SRC (H), and TNF (I). (J–L) Stigmasterol complexes with IL-6 (J), SRC (K), and TNF (L). (M–O) Ginsenoside Re complexes with IL-6 (M), SRC (N), and TNF (O). RMSF values represent residue-level flexibility and were used to assess local structural dynamics of the proteins.

We investigated the residue-level flexibility and local conformational dynamics. Root mean square fluctuation (RMSF) analysis of protein backbone atoms was performed for IL-6, SRC, and TNF in complex with selected *P. notoginseng* compounds and the reference drug Lenvatinib (Fig. 10). RMSF profiles provide insights into the mobility of individual amino acid residues and help identify flexible and stable regions within the protein structure, and ligand RMSF profiles are presented in Fig. 6, available as supplementary data at *Bioinformatics Advances* online. The Lenvatinib-bound complexes (Fig. 10a–c) exhibited moderate residue-level fluctuations, with noticeable peaks in specific regions of IL-6 and SRC, while TNF showed comparatively lower and more uniform fluctuations, indicating moderate structural flexibility. Compared to *P. notoginseng* compounds, Daucosterol-bound systems (Fig. 10d–f) demonstrated reduced fluctuations across most residues, particularly in IL-6 and TNF, suggesting enhanced structural stabilization upon ligand binding, although SRC displayed localized flexibility. Similarly, Panaxadiol complexes (Fig. 10g–i) generally showed stable RMSF profiles, with IL-6 and TNF exhibiting low-to-moderate fluctuations, while SRC showed slightly higher variability in specific regions without global destabilization. Stigmasterol complexes (Fig. 10j–l) show consistent fluctuation patterns, with relatively low RMSF values in IL-6 and SRC, whereas TNF showed moderate localized peaks, indicating controlled flexibility, and Ginsenoside Re systems (Fig. 10m–o) exhibited stable and well-distributed RMSF profiles, particularly for IL-6 and TNF, while SRC showed moderate but balanced fluctuations, suggesting preserved structural integrity. We further validate the dynamic stability of the protein–ligand complexes; additional analyses, including ligand RMSF, DCCM, and PCA, were performed. Ligand RMSF analysis (Fig. 6, available as supplementary data at *Bioinformatics Advances* online) showed that *P. notoginseng* compounds exhibited lower and more stable atomic fluctuations across IL-6, SRC, and TNF, indicating restricted mobility within the binding pocket compared to Lenvatinib. Consistently, DCCM analysis (Fig. 7, available as supplementary data at *Bioinformatics Advances* online) revealed predominantly moderate positive correlations with minimal anti-correlated motions, suggesting well-coordinated residue dynamics and the absence of large-scale structural disruptions upon ligand binding. Furthermore, PCA (Fig. 8, available as supplementary data at *Bioinformatics Advances* online) demonstrated well-defined, continuous conformational distributions with smooth transitions over the simulation time, indicating stable trajectory convergence and controlled conformational sampling.

Together, RMSD, RMSF, ligand RMSF, DCCM, and PCA analyses consistently demonstrate the structural stability and dynamic robustness of *P. notoginseng* compounds in complexes with key cervical cancer targets.

## 4 Discussion

In this integrative study, we employed systems pharmacology, ML, Multi-Omics, MDs simulation, and in silico simulation to investigate the therapeutic potential of *P. notoginseng* compounds against cervical cancer. Starting with a comprehensive physicochemical and pharmacokinetic profiling (Table 1), several bioactive constituents, including Ginsenoside Re3, Stigmasterol, Quercetin, Panaxadiol, D-Mannitol, and β-Sitosterol, satisfied drug-likeness criteria, showed predicted BBB permeability, and lacked hepatotoxicity. These ADMET characteristics are consistent with previously reported profiles of ginsenosides and triterpenoids derived from Panax species, which are known for low toxicity and promising oral bioavailability (Hou *et al.* 2021).

Cervical cancer transcriptome profiling (GSE63514 and GSE9750) revealed 2106 and 1998 DEGs, respectively, with 533 overlapping genes, underscoring extensive transcriptional reprogramming between tumor and normal tissues (Fig. 2a–c). The volcano plots (Fig. 2a and b) illustrate the distribution of significantly upregulated and downregulated genes based on defined statistical thresholds, highlighting extensive transcriptional alterations between cervical tumor and normal tissues (Zhang *et al.* 2025). Intersection analysis further confirmed a subset of consistently dysregulated genes across independent datasets, enhancing the robustness of the findings. Functional enrichment analyses indicate that these genes are primarily involved in critical processes, including nuclear division, chromosome segregation, mitotic progression, and DNA replication, reflecting key features of mitotic dysregulation (Fig. 2e and f). Furthermore, both GO (Fig. 2e–g) and KEGG pathway analyses (Fig. 2d) identify significant enrichment in cell cycle regulation, DNA replication, cellular senescence, and AGE-RAGE signaling pathways (Hua *et al.* 2025) highlighting pathways associated with proliferation, genomic instability, and cellular stress responses. Importantly, the convergence of these dysregulated pathways with predicted molecular targets of *P. notoginseng* suggests a therapeutic potential comparable to natural compound-based interventions reported in other malignancies (Thorpe *et al.* 2023), reinforcing its role as a promising multi-targeted modulator of cervical oncogenesis. An integrated analysis of transcriptomic data and drug-target interactions identified 21 overlapping genes (Fig. 3), representing key candidates involved in cervical cancer progression and therapeutic response. Functional enrichment indicated their roles in metabolic regulation, apoptosis, autophagy, and signaling pathways, highlighting coordinated metabolic and regulatory reprogramming in tumor development (Franco *et al.* 2026).

ML-based analysis of the GSE63514 and GSE9750 datasets (Fig. 4) further prioritized critical regulators, including MAPK10, EPHX2, CA9, CA4, and TOP2A, which are associated with tumor progression and signaling dysregulation (Tian and Huang 2025). The LR model showed strong predictive performance (AUC = 1.0 for training, 0.996 for cross-validation, and 0.833 for validation), indicating high accuracy with reasonable generalizability. These findings are consistent with previous studies employing integrative computational frameworks for biomarker discovery in cervical cancer (Vazquez *et al.* 2025). Overall, integrating transcriptomic profiling with ML provides robust validation of candidate genes and highlights their potential as therapeutic targets, particularly for multi-target agents such as *P. notoginseng*.

Our network pharmacology analysis highlighted 291 overlapping targets between *P. notoginseng* compounds and cervical cancer-associated genes, forming a densely interconnected network (Fig. 5b–d). Hub analysis identified TNF-α, SRC, and IL6 as central regulators (Xu *et al.* 2024), consistent with their established roles in sustaining tumor-promoting inflammation, angiogenesis, and proliferative signaling (Fig. 3a), echoing earlier findings that implicate these genes in cervical tumor inflammation, proliferation, and angiogenesis. Notably, previous studies have confirmed that IL6 and TNF-α axes are central to HPV-induced carcinogenesis and immune evasion, further validating our target selection (Wang *et al.* 2025). Additional nodes, including MAPK3, EGFR, BCL2, and ESR1, further underscore the multi-layered regulatory potential of *P. notoginseng* (Li *et al.* 2023b). PPI analysis revealed highly clustered interactions, indicating cooperative regulation of oncogenic signaling (Fig. 5c). Enrichment analyses demonstrated significant involvement in cancer-associated pathways such as steroid hormone biosynthesis, insulin resistance, and endocrine resistance (Fig. 5f). GO terms linked to inflammatory response, ERK1/2 cascade regulation, lipid metabolism, and calcium signaling provided mechanistic insight into the observed network regulation (Fig. 5e). Notably, MF analysis revealed enrichment in GPCR and serine/threonine kinase activity, aligning with TNF-α/IL6-mediated inflammation and SRC oncogenic signaling. Collectively, these findings suggest that *P. notoginseng* exerts therapeutic effects by simultaneously targeting multiple signaling pathways, thereby attenuating chronic inflammation, metabolic dysregulation, and tumor progression in cervical cancer.

Molecular docking analyses across IL-6 (Fig. 6), SRC (Fig. 7), and TNF-α (Fig. 8) demonstrate that Ginsenoside Re3 and stigmasterol consistently exhibit comparable predicted binding affinities with reference inhibitors sorafenib, sunitinib, and lenvatinib, suggesting their potential as multi-target modulators. In the IL-6 binding pocket (Fig. 6), Ginsenoside Re3 forms a stable network of hydrogen bonds complemented by hydrophobic interactions, indicating comparable binding energetics and structural compatibility. Comparable interaction patterns were observed for SRC (Fig. 7) and TNF-α (Fig. 8), in which both Ginsenoside Re3 and stigmasterol engage key residues within the active sites through a combination of polar and non-polar contacts, supporting stable ligand accommodation. In contrast, the reference inhibitors sorafenib, sunitinib, and lenvatinib generally showed slightly weaker binding affinities and fewer interactions across the three proteins. These findings are consistent with previous reports highlighting the anti-inflammatory and anti-cancer properties of Ginsenoside Re3 and phytosterols such as stigmasterol (Valdés-González *et al.* 2023). Nevertheless, molecular docking provides only an approximate estimation of binding affinity; therefore, the relatively small differences in docking scores should be interpreted qualitatively rather than as definitive rankings (Pantsar and Poso 2018). To enhance the robustness of these results, MDs simulations and interaction analyses were performed to assess binding stability over time. Further validation using more rigorous computational approaches, such as alchemical free energy calculations and enhanced sampling techniques (York 2023), will be essential to better establish the biological and therapeutic relevance of these interactions.

MDs simulations further validated the stability of identified protein–ligand interactions at an atomic level. RMSD trajectories over 200 ns revealed sustained conformational stability for *P. notoginseng* compounds Daucosterol, Panaxadiol, Stigmasterol, and Ginsenoside Re, with minimal backbone deviations indicative of stable binding and preserved structural integrity (Fig. 9). In contrast, the reference drug Lenvatinib exhibited comparatively higher fluctuations, particularly in the TNF complex, suggesting target-dependent variability in binding stability. These interaction patterns are consistent with previous MDs studies showing that natural compounds forming stable hydrogen bonds, hydrophobic contacts, and water-mediated interactions exhibit enhanced binding persistence and biological activity. Phytochemicals with sustained interaction networks and reduced fluctuations enhance anticancer efficacy by stabilizing target proteins (Aljabali *et al.* 2025). MD-based studies have highlighted that multi-type interactions and high interaction fractions are critical determinants of ligand stability and functional modulation in cancer-related proteins (Li *et al.* 2024), recent computational studies, where lupenone derivatives demonstrated stable binding dynamics against HPV-associated targets, including the E6 oncoprotein, further supporting the relevance of natural compounds as effective modulators in cervical cancer therapy (Mishra *et al.* 2025). Consistent with these findings, protein–ligand interaction analysis across IL-6, SRC, and TNF (Fig. 2, available as supplementary data at *Bioinformatics Advances* online) demonstrates that *P. notoginseng* compounds, particularly Daucosterol, Ginsenoside Re, Panaxadiol, and Stigmasterol, form stable interaction networks with higher interaction persistence compared to other ligands. Similarly, time-resolved protein–ligand interaction mapping (Fig. 3, available as supplementary data at *Bioinformatics Advances* online) further confirms that these compounds maintain consistent and persistent residue-level interactions throughout the simulation, indicating enhanced binding stability.

RMSF analysis (Fig. 10) further supported the stability of the protein–ligand complexes by showing generally reduced, well-distributed residue-level fluctuations across the IL-6, SRC, and TNF systems. Complexes involving Daucosterol, Panaxadiol, Stigmasterol, and Ginsenoside Re exhibited relatively stable RMSF profiles with only localized flexibility, indicating maintained structural integrity rather than disruption of specific SSEs.

The comprehensive MD simulation analyses, including ligand RMSF (Fig. 6, available as supplementary data at *Bioinformatics Advances* online), DCCM (Fig. 7, available as supplementary data at *Bioinformatics Advances* online), and PCA (Fig. 8, available as supplementary data at *Bioinformatics Advances* online), further support the dynamic stability and comparable interaction profiles of *P. notoginseng* compounds with key cervical cancer targets. The observed low ligand fluctuations and reduced residue-level flexibility indicate stable binding, while balanced correlation patterns and well-defined conformational spaces confirm preserved structural integrity and controlled dynamics. These findings are consistent with recent computational studies, where stable RMSF patterns were associated with effective ligand binding and anticancer activity, as demonstrated for oleocanthal-based systems targeting oncogenic pathways (Jannati *et al.* 2025). Similarly, previous analyses of the TNF-α-associated system have shown that reduced residue-level flexibility correlates with improved structural stability and functional modulation (Marzbanrad *et al.* 2024). Collectively, the observed RMSF analysis confirms the stability of *P. notoginseng* compounds in maintaining protein conformational integrity, supporting their potential as effective modulators of key cervical cancer targets.

Secondary structure analysis (Figs 4 and 5, available as supplementary data at *Bioinformatics Advances* online) further supported structural conservation upon ligand binding. The IL6-Ginsenoside Re3 complex retained more than 85% of its initial α-helix content over 200 ns, indicating strong structural stability. Similar behavior was observed for SRC and TNF-α when bound to Stigmasterol and Quercetin, respectively. These findings align with reports that stable secondary structures enhance the pharmacological compatibility of natural product protein interactions, reinforcing the potential of these compounds for therapeutic development (Othman *et al.* 2025).

Detailed protein–ligand interaction profiling from supplementary analyses (Fig. 2, available as supplementary data at *Bioinformatics Advances* online) reinforced these observations. Previous MDs studies provide strong support for our findings. As multi-dynamics simulations of ginsenosides such as Rg3 and Re3 with the receptor ADGRG3 demonstrated notably stable binding profiles, characterized by low RMSD values and consistently high hydrogen-bond occupancy throughout the simulation (Lu 2025). Similarly, MD analyses of GSK3β-inhibitor complexes identified a persistent water-mediated hydrogen bond bridge that played a crucial role in maintaining binding-site integrity (Jitendra Joshi and Raja Sekhar Reddy 2024). In addition, docking-coupled MD studies of ginsenoside FoxO3a receptor complexes revealed the presence of robust hydrophobic interactions within a defined pocket alongside multiple long-lasting hydrogen bonds, collectively contributing to enhanced complex stability in silico (Li *et al.* 2023a). In comparison to our study, the Ginsenoside Rg3, Panaxadiol, and Ginsenoside Re3 exhibited persistent multi-type interactions, including hydrogen bonding, hydrophobic contacts, ionic interactions, and water bridges across the MD timescale. Notably, Panaxadiol and Rg3 maintained continuous multi-residue contacts with SRC’s catalytic and regulatory domains (Figs 2 and 3, available as supplementary data at *Bioinformatics Advances* online), while Ginsenoside Re3 and Quercetin engaged TNF-α with high-frequency hydrogen bonds and water-mediated contacts (Figs 2 and 3, available as supplementary data at *Bioinformatics Advances* online). In IL6 complexes, Rg3 and Stigmasterol demonstrated strong hydrophobic and water-bridge contributions (Figs 2 and 3, available as supplementary data at *Bioinformatics Advances* online), indicating stable and diverse binding modes.

In summary, *P. notoginsen* phytochemicals exhibited comparable pharmacokinetic properties, stable interactions with IL-6, SRC, and TNF-α, and preserved protein structural integrity. Integration of differential gene expression and ML analyses further highlighted their involvement in key oncogenic pathways and identified 34 robust hub genes, including CA4, MAPK10, and TOP2A. These findings support advancing Ginsenoside Re3, Ginsenoside Rg3, and Panaxadiol for preclinical evaluation in HPV-positive cervical cancer models, with particular focus on their modulation of IL-6, TNF-α, and SRC signaling pathways.

## 5 Conclusion

*Panax notoginseng* phytochemicals exhibit strong multitarget therapeutic potential against cervical cancer by modulating key oncogenic and inflammatory proteins, including IL-6, TNF-α, and SRC. Key compounds such as Ginsenosides Re, Stigmasterol, Daucosterol, Panaxadiol, and Beta-sitosterol show stable interactions and predictive biological activity, with ginsenosides exhibiting performance comparable to reference inhibitors. Collectively, these findings support *P. notoginseng* as a promising natural source for developing novel therapeutics targeting cervical cancer progression.

## Supplementary Material

vbag164_Supplementary_Data

## Data Availability

All datasets generated for this study are available from the corresponding authors on reasonable request.
