# Supplementary material for: Exploring the molecular basis of Panax notoginseng mediated inhibition of cervical cancer through machine learning, transcriptomics analysis, network pharmacology, molecular docking, and in-silico simulation approaches
Source: Bioinform Adv. 2026 Jul 10;6(1):vbag164. doi: 10.1093/bioadv/vbag164 (PMC13458595; doi:10.1093/bioadv/vbag164)
Supplement: Supplementary file 1 [file vbag164_supplementary_data.zip › Supplementary Data.docx]

**Supporting Information for**

**Exploring the Molecular Basis of *Panax-notoginseng* Mediated Inhibition of Cervical Cancer through Machine Learning, Transcriptomics Analysis, Network Pharmacology, Molecular Docking, and *In-Silico* Simulation Approaches**

Shah Kamal, ^1,2,3^*, Chen Qin ^1,2,3^*, Yanjuan Wang ^1,2,3^, Ruilin He ^1,2,3^, Mohammad Amjad Kamal ^4,5,^ and Wenji Li ^1,2,3#^

^1^School of Traditional Chinese Medicine, Faculty of Medicine, Yangzhou University, Yangzhou, 225009, Jiangsu, China

^2^Key Laboratory of the Jiangsu Higher Education Institutions for Integrated Traditional Chinese and Western Medicine in Senile Diseases Control (Yangzhou University), Yangzhou, 225009, Jiangsu, China

^3^Sino-Malaysia Molecular Oncology and Traditional Chinese Medicine Delivery Joint Research Centre, Medical College, Yangzhou University, Yangzhou, 225009, jiangsu, China

^4^Future Technologies Research Centre, King Faisal University, Al Ahsa, PO Box 400 - Post Code 31982, Saudi Arabia

^5^Novel Global Community Educational Foundation, Australia

*These authors contributed equally to this work.

^#^Corresponding author: Wenji Li, Institute of Translational Medicine, Medical College, Yangzhou University, Yangzhou, Jiangsu 225009, PR. China. Tel: +86-13222680359 E-mail: (Wenji_li@yzu.edu.cn).

**Table S1.** Target Search Databases

| **Full Name** | **Abbreviation** | **URL** | **Access time** |
| --- | --- | --- | --- |
| Traditional Chinese Medicine Systems Pharmacology Database and Analysis Platform | TCMSP | http://tcmsp-e.com/ | March 20, 2025 |
| The Human Genes Compendium | GeneCards | https://www.genecards.org/ | March 21, 2025 |
| Swiss Absorption, Distribution, Metabolism, and Excretion | SwissADME | http://www.swissadme.ch/ | April 20, 2025 |
| SwissTargetPrediction | SwissADME | <http://www.swisstargetprediction.ch/> | April 25, 2025 |
| Venny`s diagram 2.1 | \ | <https://bioinfogp.cnb.csic.es/tools/venny/> | April 26, 2025 |
| Search Tool for the Retrieval of Interacting Genes | STRING | <https://string-db.org/>; | May 5, 2025 |
| Database for Annotation, Visualization, and Integrated Discovery | DAVID | <https://david.ncifcrf.gov>; | May 16, 2025 |
| PubChem | \ | <https://pubchem.ncbi.nlm.nih.gov> | June 12, 2025 |
| Protein Data Bank | PDB | <https://www.rcsb.org/> | June 24, 2025 |

**Table S2.** Binding Energies of Compounds with Disease Targets

| **Name of Compounds** | **Name of Targets** | **Binding affinity (kcal/mol)** |
| --- | --- | --- |
| Sorafenib | IL6 | −10.0 |
| Sunitinib | IL6 | −9.6 |
| Lenvatinib | IL6 | −9.5 |
| Stigmasterol | IL6 | −10.9 |
| Daucosterol | IL6 | −10.8 |
| Ginsenoside Re | IL6 | −10.4 |
| Panaxadiol | IL6 | −10.2 |
| D-Mannitol | IL6 | −5.4 |
| β-sitosterol | IL6 | −9.8 |
| Ginsenoside Re3 | IL6 | −9.0 |
| Quercetin | IL6 | -7.9 |
| Sorafenib | SRC | −10.4 |
| Sunitinib | SRC | −9.1 |
| Lenvatinib | SRC | −9.3 |
| Panaxadiol | SRC | −10.4 |
| Ginsenoside Re | SRC | −10.2 |
| Stigmasterol | SRC | −10.0 |
| Daucosterol | SRC | −10.0 |
| β-Sitosterol | SRC | −9.6 |
| Quercetin | SRC | −9.3 |
| Ginsenoside Re3 | SRC | −9.9 |
| D-Mannitol | SRC | −5.5 |
| Sorafenib | TNF | −8.6 |
| Sunitinib | TNF | −7.7 |
| Lenvatinib | TNF | −8.4 |
| Daucosterol | TNF | −9.1 |
| stigmasterol | TNF | −8.8 |
| quercetin | TNF | −8.8 |
| panaxadiol | TNF | −8.6 |
| ginsenoside Re | TNF | −8.6 |
| ginsenoside Re3 | TNF | −8.2 |
| β-Sitosterol | TNF | −7.9 |
| D-mannitol | TNF | −5.3 |

**Fig. S1. Functional Enrichment analysis shows hormonal, metabolic, inflammatory and oncogenic pathways.**

**Fig. S2.** **Residue-level interaction fractions of IL-6, SRC, and TNF with *Panax-notoginseng* phytochemicals and the control drug Lenvatinib over 200 ns molecular dynamics simulations.** Interaction fractions represent the proportion of simulation time during which specific residues participate in hydrogen bonds, hydrophobic contacts, ionic interactions, and water bridges. (A–C) Lenvatinib complexes with IL-6 (A), SRC (B), and TNF (C). (D–F) Daucosterol complexes with IL-6 (D), SRC (E), and TNF (F). (G–I) Panaxadiol complexes with IL-6 (G), SRC (H), and TNF (I). (J–L) Stigmasterol complexes with IL-6 (J), SRC (K), and TNF (L). (M–O) Ginsenoside Re complexes with IL-6 (M), SRC (N), and TNF (O). Higher interaction fractions indicate more persistent residue-level interactions contributing to complex stability.

**Fig. S3. Time-resolved residue-level interaction mapping of IL-6, SRC, and TNF with *Panax-notoginseng* phytochemicals and the control drug Lenvatinib over 200 ns molecular dynamics simulations.** The upper panels represent the total number of contacts formed over time, while the lower heatmaps illustrate residue-specific interaction persistence throughout the simulation. (A–C) Lenvatinib complexes with IL-6 (A), SRC (B), and TNF (C). (D–F) Daucosterol complexes with IL-6 (D), SRC (E), and TNF (F). (G–I) Panaxadiol complexes with IL-6 (G), SRC (H), and TNF (I). (J–L) Stigmasterol complexes with IL-6 (J), SRC (K), and TNF (L). (M–O) Ginsenoside Re complexes with IL-6 (M), SRC (N), and TNF (O). Darker color intensity indicates higher interaction frequency and persistence over the simulation period.

**Fig. S4. Secondary structure element (SSE) distribution of IL-6, SRC, and TNF in complex with *Panax-notoginseng* phytochemicals and the control drug Lenvatinib.** The plots show the percentage of secondary structural elements (%SSE), including α-helices and β-strands, as a function of the residue index. (A–C) Lenvatinib complexes with IL-6 (A), SRC (B), and TNF (C). (D–F) Daucosterol complexes with IL-6 (D), SRC (E), and TNF (F). (G–I) Panaxadiol complexes with IL-6 (G), SRC (H), and TNF (I). (J–L) Stigmasterol complexes with IL-6 (J), SRC (K), and TNF (L). (M–O) Ginsenoside Re complexes with IL-6 (M), SRC (N), and TNF (O). Consistent secondary structure patterns indicate preservation of protein structural integrity upon ligand binding.

**Fig. S5. Dynamic secondary structure evolution of IL-6, SRC, and TNF during 200 ns molecular dynamics simulations in complex with *Panax-notoginseng* phytochemicals and the control drug Lenvatinib.** The upper panels represent the overall percentage of secondary structure content (%SSE) over time, while the lower panels show residue-wise secondary structure transitions throughout the simulation. (A–C) Lenvatinib complexes with IL-6 (A), SRC (B), and TNF (C). (D–F) Daucosterol complexes with IL-6 (D), SRC (E), and TNF (F). (G–I) Panaxadiol complexes with IL-6 (G), SRC (H), and TNF (I). (J–L) Stigmasterol complexes with IL-6 (J), SRC (K), and TNF (L). (M–O) Ginsenoside Re complexes with IL-6 (M), SRC (N), and TNF (O). Stable secondary structure patterns over time indicate maintained structural integrity of the protein–ligand complexes.

**Fig. S6. Root mean square fluctuation (RMSF) analysis of ligands in protein-ligand complexes.** RMSF profiles of ligand atoms for complexes of IL-6, SRC, and TNF with *Panax-notoginseng* compounds and the reference drug Lenvatinib over 200 ns molecular dynamics simulations. (A–C) Lenvatinib complexes with IL-6 (A), SRC (B), and TNF (C). (D–F) Daucosterol complexes with IL-6 (D), SRC (E), and TNF (F). (G–I) Panaxadiol complexes with IL-6 (G), SRC (H), and TNF (I). (J–L) Stigmasterol complexes with IL-6 (J), SRC (K), and TNF (L). (M–O) Ginsenoside Re complexes with IL-6 (M), SRC (N), and TNF (O). RMSF values represent ligand atomic fluctuations and were used to assess ligand flexibility and stability within the binding pocket.

**Fig. S7. Dynamic cross-correlation matrix (DCCM) analysis of protein-ligand complexes.** Residue–residue cross-correlation maps derived from molecular dynamics simulations for IL-6, SRC, and TNF in complex with the reference drug Lenvatinib and *Panax-notoginseng* compounds. (A–C) Lenvatinib complexes with IL-6 (A), SRC (B), and TNF (C). (D–F) Daucosterol complexes with IL-6 (D), SRC (E), and TNF (F). (G–I) Panaxadiol complexes with IL-6 (G), SRC (H), and TNF (I). (J–L) Stigmasterol complexes with IL-6 (J), SRC (K), and TNF (L). (M–O) Ginsenoside Re complexes with IL-6 (M), SRC (N), and TNF (O). Positive correlations (blue) indicate coordinated residue motions, while negative correlations (light colors) represent anti-correlated movements. The diagonal represents self-correlation of residues.

**Fig. S8. Principal component analysis (PCA) of protein-ligand complexes.** Projection of molecular dynamics trajectories onto the first two principal components (PC1 and PC2) for IL-6, SRC, and TNF in complex with the reference drug Lenvatinib and *Panax-notoginseng* compounds. (A-C) Lenvatinib complexes with IL-6 (A), SRC (B), and TNF (C). (D-F) Daucosterol complexes with IL-6 (D), SRC (E), and TNF (F). (G-I) Panaxadiol complexes with IL-6 (G), SRC (H), and TNF (I). (J-L) Stigmasterol complexes with IL-6 (J), SRC (K), and TNF (L). (M-O) Ginsenoside Re complexes with IL-6 (M), SRC (N), and TNF (O). Each point represents a simulation frame, colored by simulation time (red to blue), illustrating conformational space sampling and the dynamic stability of the complexes.
