# Supplementary material for: Exploring the molecular basis of Panax notoginseng mediated inhibition of cervical cancer through machine learning, transcriptomics analysis, network pharmacology, molecular docking, and in-silico simulation approaches
Source: Bioinform Adv. 2026 Jul 10;6(1):vbag164. doi: 10.1093/bioadv/vbag164 (PMC13458595; doi:10.1093/bioadv/vbag164)
Supplement: Supplementary file 1 [file vbag164_supplementary_data.zip › Table S1.docx]

**Table S1.** Target Search Databases

| **Full Name** | **Abbreviation** | **URL** | **Access time** |
| --- | --- | --- | --- |
| Traditional Chinese Medicine Systems Pharmacology Database and Analysis Platform | TCMSP | http://tcmsp-e.com/ | March 20, 2025 |
| The Human Genes Compendium | GeneCards | https://www.genecards.org/ | March 21, 2025 |
| Swiss Absorption, Distribution, Metabolism, and Excretion | SwissADME | http://www.swissadme.ch/ | April 20, 2025 |
| SwissTargetPrediction | SwissADME | <http://www.swisstargetprediction.ch/> | April 25, 2025 |
| Venny`s diagram 2.1 | \ | <https://bioinfogp.cnb.csic.es/tools/venny/> | April 26, 2025 |
| Search Tool for the Retrieval of Interacting Genes | STRING | <https://string-db.org/>; | May 5, 2025 |
| Database for Annotation, Visualization, and Integrated Discovery | DAVID | <https://david.ncifcrf.gov>; | May 16, 2025 |
| PubChem | \ | <https://pubchem.ncbi.nlm.nih.gov> | June 12, 2025 |
| Protein Data Bank | PDB | <https://www.rcsb.org/> | June 24, 2025 |
