# Supplementary material for: Exploring the molecular basis of Panax notoginseng mediated inhibition of cervical cancer through machine learning, transcriptomics analysis, network pharmacology, molecular docking, and in-silico simulation approaches
Source: Bioinform Adv. 2026 Jul 10;6(1):vbag164. doi: 10.1093/bioadv/vbag164 (PMC13458595; doi:10.1093/bioadv/vbag164)
Supplement: Supplementary file 1 [file vbag164_supplementary_data.zip › Table S2.docx]

**Table S2.** Binding Energies of Compounds with Disease Targets

| **Name of Compounds** | **Name of Targets** | **Binding affinity (kcal/mol)** |
| --- | --- | --- |
| Sorafenib | IL6 | −10.0 |
| Sunitinib | IL6 | −9.6 |
| Lenvatinib | IL6 | −9.5 |
| Stigmasterol | IL6 | −10.9 |
| Daucosterol | IL6 | −10.8 |
| Ginsenoside Re | IL6 | −10.4 |
| Panaxadiol | IL6 | −10.2 |
| D-Mannitol | IL6 | −5.4 |
| β-sitosterol | IL6 | −9.8 |
| Ginsenoside Re3 | IL6 | −9.0 |
| Quercetin | IL6 | -7.9 |
| Sorafenib | SRC | −10.4 |
| Sunitinib | SRC | −9.1 |
| Lenvatinib | SRC | −9.3 |
| Panaxadiol | SRC | −10.4 |
| Ginsenoside Re | SRC | −10.2 |
| Stigmasterol | SRC | −10.0 |
| Daucosterol | SRC | −10.0 |
| β-Sitosterol | SRC | −9.6 |
| Quercetin | SRC | −9.3 |
| Ginsenoside Re3 | SRC | −9.9 |
| D-Mannitol | SRC | −5.5 |
| Sorafenib | TNF | −8.6 |
| Sunitinib | TNF | −7.7 |
| Lenvatinib | TNF | −8.4 |
| Daucosterol | TNF | −9.1 |
| stigmasterol | TNF | −8.8 |
| quercetin | TNF | −8.8 |
| panaxadiol | TNF | −8.6 |
| ginsenoside Re | TNF | −8.6 |
| ginsenoside Re3 | TNF | −8.2 |
| β-Sitosterol | TNF | −7.9 |
| D-mannitol | TNF | −5.3 |
